# *TOMM40‘523’* poly-T repeat length is a determinant of longitudinal cognitive decline in Parkinson’s disease

**DOI:** 10.1038/s41531-021-00200-y

**Published:** 2021-07-07

**Authors:** Megan C. Bakeberg, Anastazja M. Gorecki, Abigail L. Pfaff, Madison E. Hoes, Sulev Kõks, P. Anthony Akkari, Frank L. Mastaglia, Ryan S. Anderton

**Affiliations:** 1 Perron Institute for Neurological and Translational Science, Nedlands, WA Australia; 2 Centre for Neuromuscular and Neurological Disorders, University of Western Australia, Nedlands, WA Australia; 3 School of Biological Sciences, University of Western Australia, Crawley, WA Australia; 4 The Centre for Molecular Medicine and Innovative Therapeutics, Murdoch University, Murdoch, WA Australia; 5 Institute for Health Research and School of Health Sciences, University of Notre Dame Australia, Fremantle, WA Australia

**Keywords:** Parkinson's disease, Predictive markers, Genotype, Parkinson's disease

## Abstract

The translocase of outer mitochondrial membrane 40 (*TOMM40*) *‘523’* polymorphism has previously been associated with age of Alzheimer’s disease onset and cognitive functioning in non-pathological ageing, but has not been explored as a candidate risk marker for cognitive decline in Parkinson’s disease (PD). Therefore, this longitudinal study investigated the role of the *‘523’* variant in cognitive decline in a patient cohort from the Parkinson’s Progression Markers Initiative. As such, a group of 368 people with PD were assessed annually for cognitive performance using multiple neuropsychological protocols, and were genotyped for the *TOMM40 ‘523’* variant using whole-genome sequencing data. Covariate-adjusted generalised linear mixed models were utilised to examine the relationship between *TOMM40 ‘523’* allele lengths and cognitive scores, while taking into account the *APOE* ε genotype. Cognitive scores declined over the 5-year study period and were lower in males than in females. When accounting for *APOE* ε4, the *TOMM40 ‘523’* variant was not robustly associated with overall cognitive performance. However, in *APOE* ε3/ε3 carriers, who accounted for ~60% of the whole cohort, carriage of shorter *‘523’* alleles was associated with more severe cognitive decline in both sexes, while carriage of the longer alleles in females were associated with better preservation of global cognition and a number of cognitive sub-domains, and with a delay in progression to dementia. The findings indicate that when taken in conjunction with the *APOE* genotype, *TOMM40 ‘523’* allele length is a significant independent determinant and marker for the trajectory of cognitive decline and risk of dementia in PD.

## Introduction

Cognitive impairment is a notable symptom of Parkinson’s disease (PD), not only because of its impact on patient-perceived quality of life, but also because significant dysfunction will affect a high proportion of people with PD (PwP) as the disease progresses^1,2^. Past research has reported a marked degree of clinical heterogeneity and different patterns of brain pathology have been identified as the underlying basis for cognitive decline in PD^3–5^, suggestive of a variable underlying pathophysiology and a complex interaction between different risk factors, including genetic variability. Candidate genes may account for a portion of susceptibility to cognitive impairment in PwP, risk of Parkinson’s disease dementia (PDD), and for the pattern of decline within certain cognitive domains and rate of decline^6–8^.

Prior candidate genetic studies and genome-wide association studies have been central in identifying genetic factors that contribute to the development of cognitive dysfunction in PD, in particular the significant role of Apolipoprotein E Epsilon 4 (*APOE ε4*) in modulating risk of cognitive decline^9–11^. However, alternative approaches, including analysis of structural variants^12^ and variation within noncoding regions of the genome^13,14^ may also help to elucidate complex phenotypic heterogeneity^15^. One structural variant of note is the ‘translocase of outer mitochondrial membrane 40’ (*TOMM40*) polymorphic T-tract length (rs10524523, ‘*523’*); an intronic variant known to improve precision when estimating the age of onset of Alzheimer’s disease (AD) in *APOE* ε3 carriers and to be associated with changes in cognition in non-pathological ageing^16–22^. While the functional effects of polymorphism at the *‘523’* locus remain largely unknown, a reduction in the TOMM40 protein due to the decreased *TOMM40* expression is known to be associated with α-synuclein accumulation and a number of other effects, including increased reactive oxygen species formation, oxidative damage, reduced mitochondrial integrity and neuroinflammation^23–27^. In view of the purported functional impact of allelic variation at the ‘*523’* locus, coupled with the shared clinical, pathological and molecular features of AD and PD^28–30^, polymorphism at this site is therefore a plausible candidate to modulate the development and progression of cognitive impairment in PD. Though, it is important to note that consideration of the effect of *APOE* must be taken into account, as previously reported^16^.

Our group, along with others, have previously demonstrated that polymorphism at the *TOMM40 ‘523’* locus is not a determinant of PD risk, although it may be a modifier of the age of symptom onset^31–33^. However, studies examining the possible role of ‘*523’* alleles in modulating cognitive decline in PD are fundamentally lacking, with only a single cross-sectional study reporting a lack of association with dementia in a mixed PD and diffuse Lewy body disease (DLB) cohort^34^. Furthermore, with sexual dimorphism being an increasingly reported phenomenon in the context of both cognition^35–37^, as well as in the cognitive status of people with PD^38–40^, it is imperative to consider such factors. As such, the primary objective of the present study was to examine the role of *TOMM40* ‘523’ alleles as determinants of the severity and trajectory of cognitive decline in a large, well-documented Caucasian PD cohort, and to determine whether the alleles are associated with differential effects on specific cognitive domains, when taking patient sex into account.

## Results

### Baseline demographic and cognitive performance data

Baseline and longitudinal clinical and demographic information and cognitive scores for the 368 participants are presented in Supplementary Tables 2 and 3. At baseline, the majority of participants were males (65.5%), had an average age of PD symptom onset of 59.85 (±9.73) years, with a disease duration of 6.76 (±6.65) years, a mean age at the time of assessment of 61.79 (±9.56) years, and a mean education duration of 15.55 (±2.94) years. Cognitive assessment scores at baseline were Montreal Cognitive Assessment (MoCA) 27.22 (±2.24) points, Hopkins Verbal Learning Test-Revised (HVLT) 24.46 (±4.94) points, Benton Judgement of Line Orientation (BJLO) 12.85 (±2.07) points, Semantic Fluency test—COMbined (SFCOM) 48.87 (±11.76) points, Letter–Number Sequencing (LNS) 10.71 (±2.62) points and Symbol Digit Modalities Test (SDMT) 41.08 (±9.53) points. Within this group, repeated measures analyses revealed significant decline of the MoCA (*p* = 0.004), LNS (*p* = 0.011) and SDMT (*p* = 0.044) scores over the study period, while HVLT (*p* = 0.598), BJLO (*p* = 0.710) and SFCOM (*p* = 0.499) scores were not altered significantly (Supplementary Table 4).

### *TOMM40 ‘523’* alleles and longitudinal cognitive performance in whole cohort

As aforementioned, allelic and genotypic distributions of *TOMM40 ‘523’* are presented in Supplementary Table 1, with 45.2% of the 368 participants possessing the *‘523’* S allele, 10.7% the L allele and 44% carrying the VL allele.

Subsequently, in adjusted generalised linear mixed models (GLMMs), carriage of S alleles was associated with a significantly greater decline in the MoCA score over the study period (*p* = 0.009; Bonferroni corrected *p* = 0.036), and decline in HVLT (*p* = 0.017), and SFCOM scores (*p* = 0.001; Bonferroni corrected *p* = 0.004; Table 1), though the decline in HVLT did not remain statistically significant after Bonferroni correction. Conversely, carriage of L alleles was associated with superior scoring in the BJLO and LNS tests over the study period (*p* < 0.001 and *p* < 0.001, respectively; Bonferroni corrected *p* = 0.004 and *p* = 0.004, respectively). In unadjusted linear models, S and VL alleles were associated with better performance in the LNS (*p* = 0.015) and SDMT tests (*p* = 0.012), respectively, but these associations were not confirmed after adjusted for covariates and multiple comparison correction (Table 1).

**Table 1:** Differential effects of *TOMM40 ‘523’* alleles in predicting cognitive performance over time, using unadjusted and adjusted generalised linear mixed models (*n* = 368).

		Unadjusted					Adjusted				
Model	Outcome	Intercept	β-CoE	SE	*t* Value	*p*^b^ Value	Intercept	β-CoE	SE	*t* value	*p*^c^ Value
	MoCA	26.827	−0.098	0.144	−0.679	0.497	31.953	−0.376	0.144	−2.610	**0.009**
	HVLT	24.367	−0.108	0.282	−0.384	0.701	33.208	−0.658	0.276	−2.380	**0.017**
S present^a^	BJLO	12.643	0.155	0.109	1.432	0.152	13.973	0.073	0.112	0.650	0.516
(*n* = 333)	SFCOM	48.922	−0.172	0.619	−0.278	0.781	57.851	−1.892	0.592	−3.194	0.**001**
	LNS	10.184	0.349	0.143	2.440	**0.015**	15.489	0.164	0.142	1.159	0.247
	SDMT	40.498	−0.059	0.546	−0.108	0.914	63.680	−0.950	0.517	−1.837	0.066
	MoCA	26.767	−0.052	0.157	−0.331	0.740	31.677	0.112	0.318	0.353	0.724
	HVLT	24.287	0.012	0.306	0.039	0.969	33.053	0.535	0.614	0.872	0.384
L present^a^	BJLO	12.769	−0.071	0.118	−0.602	0.547	14.207	0.927	0.247	3.750	**<0.001**
(*n* = 79)	SFCOM	48.697	0.458	0.673	0.681	0.496	61.144	2.122	1.315	1.614	0.107
	LNS	10.373	0.275	0.156	1.765	0.078	15.575	1.126	0.312	3.607	**<0.001**
	SDMT	40.476	−0.092	0.593	−0.155	0.877	65.906	1.024	1.145	0.894	0.372
	MoCA	26.757	−0.001	0.141	−0.001	0.994	31.663	0.020	0.136	0.145	0.885
	HVLT	23.985	0.443	0.274	1.615	0.106	32.746	0.371	0.261	1.422	0.155
VL present^a^	BJLO	12.816	−0.091	0.106	−0.856	0.391	14.315	−0.109	0.106	−1.033	0.302
(*n* = 116)	SFCOM	48.665	0.193	0.602	0.321	0.748	61.107	0.080	0.559	0.144	0.886
	LNS	10.437	−0.005	0.140	−0.039	0.969	15.626	−0.035	0.133	−0.263	0.793
	SDMT	39.540	1.329	0.531	2.501	**0.012**	65.463	0.554	0.488	1.137	0.256

The numbers in bold are indicative of statistical significance.

*S* short, *L* long, *VL* very long, *MoCa* Montreal Cognitive Assessment, *HVLT* Hopkins Verbal Learning Test-Revised, *BJLO* Benton Judgement of Line Orientation, *SFCOM* Semantic Fluency test—COMbined, *LNS* the Letter–Number Sequencing, *SDMT* Symbol Digit Modalities Test, *β-CoE* beta coefficient, *SE* standard error, *p* statistical significance (*p* value).

^a^Comparison category set to zero.

^b^*p* Value taken from GLMM without adjustment for covariates.

^c^*p* Value taken from GLMM adjusting for *APOE* ε4 status, as well as covariates identified in Supplementary Table 4, including years between assessments, age at assessment, age at disease onset, disease duration, gender and years of education.

### *TOMM40 ‘523’* alleles and cognitive performance in *APOE* ε3/ε3 carriers

As previous literature has indicated that the predictive value of *TOMM40 ‘523’* in late-onset AD primarily applies to carriers of *APOE* ε3, analyses were conducted again within the dominant sub-group of *APOE* ε3/ε3 carriers (*n* = 205). In this group, unadjusted linear models also demonstrated that presence of the S alleles was associated with more severe global cognitive decline (*p* < 0.001; Bonferroni corrected *p* = 0.004), as measured by the MoCA, and with more severe decline in HVLT (*p* = 0.044), SFCOM (*p* = 0.009; Bonferroni corrected *p* = 0.036) and SDMT (*p* = 0.001; Bonferroni corrected *p* = 0.004) scores over time (Table 2), though decline in HLVT did not remain statistically significant after Bonferroni correction. These associations were confirmed in covariate-adjusted models: MoCA (*p* < 0.001), SFCOM (*p* = 0.03) and SDMT (*p* = 0.022; Table 2).

**Table 2:** Capacity of *TOMM40* alleles in predicting cognitive performance over time, using unadjusted and adjusted generalised linear mixed models, in the sub-group of *APOE* ε3/ε3 carriers within the PPMI cohort.

		Unadjusted					Adjusted				
Model	Outcome	Intercept	β-CoE	SE	*t* Value	*p*^b^ Value	Intercept	β-CoE	SE	*t* Value	*p*^c^ Value
	MoCA	27.365	−0.710	0.198	−3.576	**<0.001**	31.108	−0.661	0.189	−3.506	**<0.001**
	HVLT	25.092	−0.812	0.403	−2.015	**0.044**	31.227	−0.629	0.372	−1.692	0.091
S present^a^	BJLO	12.747	0.068	0.158	0.431	0.667	12.733	0.040	0.153	0.261	0.795
(*n* = 219)	SFCOM	51.288	−2.200	0.835	−2.635	**0.009**	54.430	−1.665	0.792	−2.102	**0.036**
	LNS	10.448	0.017	0.195	0.085	0.932	14.199	0.026	0.187	0.137	0.891
	SDMT	43.064	−2.477	0.745	−3.324	**0.001**	58.218	−1.522	0.662	−2.300	**0.022**
	MoCA	26.564	0.338	0.181	1.864	0.063	30.692	0.042	0.172	0.245	0.806
	HVLT	23.527	1.286	0.365	3.524	**<0.001**	30.391	0.463	0.338	1.370	0.171
VL present^a^	BJLO	12.779	0.029	0.143	0.206	0.837	12.684	0.068	0.138	0.488	0.626
(*n* = 189)	SFCOM	48.624	1.299	0.759	1.713	0.087	53.685	−0.136	0.719	−0.190	0.850
	LNS	10.112	0.487	0.176	2.769	**0.006**	13.960	0.237	0.169	1.401	0.161
	SDMT	38.848	3.158	0.674	4.687	**<0.001**	55.592	1.702	0.600	2.835	**0.005**

The numbers in bold are indicative of statistical significance.

*S* short, *L* long, *VL* very long, *MoCa* Montreal Cognitive Assessment, *HVLT* Hopkins Verbal Learning Test-Revised, *BJLO* Benton Judgement of Line Orientation, *SFCOM* Semantic Fluency test—COMbined, *LNS* the Letter–Number Sequencing, *SDMT* Symbol Digit Modalities Test, *β-CoE* beta coefficient, *SE* standard error, *p* statistical significance (*p* value).

^a^Comparison category set to zero.

^b^*p* Value taken from GLMM without adjusting for covariates.

^c^*p* Value taken from GLMM adjusting for covariates identified in Supplementary Table 4, including years between assessments, age at assessment, age at disease onset, disease duration, gender and years of education.

In contrast, carriage of longer *‘523’* alleles were associated with preservation of cognitive performance in a number of domains. Unadjusted modelling disclosed a positive association between VL alleles and HVLT (*p* < 0.001), LNS (*p* = 0.006) and SDMT (*p* < 0.001) scores over time (Table 2). After allowing for covariates, only the association between VL alleles and better performance in the SDMT test remained significant (*p* = 0.005; Bonferroni corrected *p* = 0.020).

### Sex-specific effects of *TOMM40 ‘523’* alleles on longitudinal cognitive performance in *APOE* ε3/ε3 carriers

As previous literature has indicated that cognitive ability and cognitive decline significantly differ between males and females with PD^40^, analyses were conducted again considering the sexes as separate groups. The results are summarised in Table 3 and in Supplementary Tables 5 and 6. While in males, unadjusted GLMMs revealed a number of significant associations between *‘523’* alleles and cognitive function, only the association between carriage of S alleles and more severe decline in the MoCA remained significant after adjusted for covariates (*p* = 0.011; Table 3).

**Table 3:** Summary of differential associations of *TOMM40* alleles with longitudinal cognitive measures in male and female *APOE* ε3/ε3 carriers in the PPMI cohort.

	S allele^a^ (*n* = 219)				VL allele^a^ (*n* = 189)			
Assessment	Males		Females		Males		Females	
	β-CoE	*p*^b^ Value	β-CoE	*p*^b^ Value	β-CoE	*p*^b^ Value	β-CoE	*p*^b^ Value
MoCA	−0.602	**0.011**	−0.905	**0.006**	−0.307	0.125	1.024	0.**002**
HVLT	−0.743	0.128	−0.917	0.117	0.363	0.383	0.912	0.120
BJLO	0.100	0.576	0.195	0.476	−0.168	0.269	0.542	**0.050**
SFCOM	−2.023	0.051	−1.958	0.123	−1.697	0.053	4.235	**0.001**
LNS	−0.141	0.561	0.306	0.315	0.035	0.867	0.687	**0.024**
SDMT	−1.409	0.099	−1.815	0.094	1.304	0.073	2.543	**0.020**

The numbers in bold are indicative of statistical significance.

*S* short, *L* long, *VL* very long, *MoCa* Montreal Cognitive Assessment, *HVLT* Hopkins Verbal Learning Test-Revised, *BJLO* Benton Judgement of Line Orientation, *SFCOM* Semantic Fluency test—COMbined, *LNS* the Letter–Number Sequencing, *SDMT* Symbol Digit Modalities Test, *β-CoE* beta coefficient, *SE* standard error, *p* statistical significance (*p* value).

^a^Comparison category set to zero.

^b^*p* Value taken from GLMM adjusting for covariates identified in Supplementary Table 4, including years between assessments, age at assessment, age at disease onset, disease duration and years of education.

Notably, in females a number of associations were apparent between carriage of longer *‘523’* alleles and cognitive ability over time. Unadjusted models revealed significant associations between L alleles and worse decline in the HVLT (*p* = 0.033) assessment (Supplementary Table 6), whereas carriage of VL alleles was associated with significantly better performance over time with the MoCA (*p* = 0.024), the BJLO (*p* = 0.039), SFCOM (*p* = 0.004) and LNS (*p* = 0.045) assessments (Supplementary Table 6). Multivariable GLMMs confirmed that carriage of S alleles was associated with significantly poorer performance in the MoCA over time (*p* = 0.006; Table 3; Bonferroni corrected *p* = 0.024). Finally, carriers of VL alleles had significantly better longitudinal cognitive performance with the MoCA (*p* = 0.002; Bonferroni corrected *p* = 0.008), BJLO (*p* = 0.050), SFCOM (*p* = 0.001; Bonferroni corrected *p* = 0.004), LNS (*p* = 0.024) and SDMT (*p* = 0.020) assessments, though BJLO, LNS and SDMT did not remain significant after correction for multiple comparisons.

### Effects of *TOMM40 ‘523’* alleles on progression to PDD

The full cohort was subsequently analysed to examine the effect of *‘523’* alleles on progression to PDD. As most baseline cognitive scores were found to differ significantly between males and females (Supplementary Table 2), analyses were carried out with and without stratification by sex.

Firstly, combined *‘523’* allele lengths were compared between the group with PDD and the group without dementia (PD-ND; Supplementary Table 7). When gender was combined, no significant differences were observable between the PDD and PD-ND groups (50.88 ± 14.01 and 52.01 ± 13.62, respectively). When separated by sex, combined ‘*523*’ allele length did not significantly differ in males with PDD vs PD-ND (51.66 ± 14.14 and 51.67 ± 13.82, respectively), whereas in females there was a significant difference in combined allele length between females with PDD (48.03 ± 13.29) and females with PD-ND (52.59 ± 13.22; *p* = 0.004; Bonferroni corrected *p* = 0.016), pointing to a protective effect of longer alleles in females only.

**Fig. 1:**
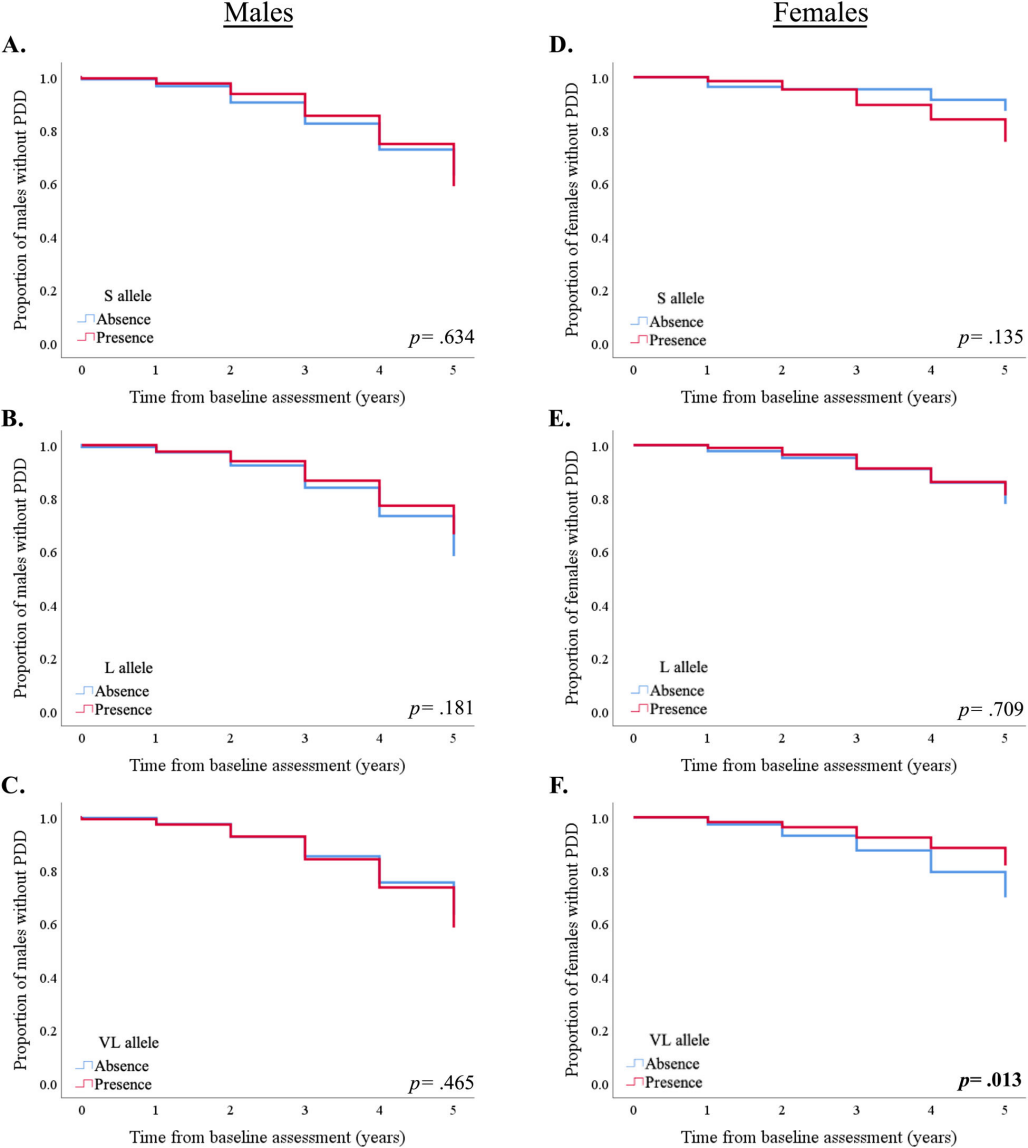
Possession of *TOMM40 ‘523’* VL allele is significantly protective against progression to PDD. Kaplan–Meier curves showing the effect of *TOMM40 ‘523’* allele length on the proportion of male participants diagnosed with PDD (S allele, **A**; L allele, **B**; and VL allele, **C**), as well as in females (**D**, **E**, **F**). PDD Parkinson’s disease dementia, S short, L long, VL very long.

In subsequent survival analyses, taking PDD diagnosis as the endpoint, no significant differences were exhibited between *‘523’* S, L or VL carrier status when the sex of the Parkinson’s Progression Markers Initiative (PPMI) cohort was combined (*p* = 0.944, *p* = 0.271 and *p* = 0.549, respectively). Analyses were re-run using Cox proportional hazard regression models, adjusting for *APOE* ε4 carrier status, and no significant associations were noted (S allele, *p* = 0.805; L allele, *p* = 0.192; VL allele, *p* = 0.759). After separating by sex, no significant associations were observable between *‘523’* allele carrier status in males, using the Kaplan–Meier method (Fig. 1A, S allele, *p* = 0.634; Fig. 1B, L allele, *p* = 0.181; Fig. 1C, VL allele, *p* = 0.465) or Cox proportional hazard regression models (S allele, *p* = 0.608; L allele, *p* = 0.889; VL allele, *p* = 0.856), which adjusted for *APOE* ε4 carrier status. In females, there were no significant observable effects with carriage of the S or L alleles using the Kaplan–Meier method, though there was a trend towards more rapid progression to dementia in females with the S allele (Fig. 1D, S allele, *p* = 0.135; Fig. 1E, L allele, *p* = 0.709). Cox proportional hazard regression found no significant effect when adjusting for *APOE* ε4 carrier status (S allele, *p* = 0.218; L allele, *p* = 0.924). Notably, a significantly faster progression to dementia was apparent in females who did not carry VL alleles (Fig. 1F, VL allele, *p* = 0.033). This association remained significant when using Cox proportional hazard regression (*p* = 0.013) and after adjusting for carriage of *APOE* ε4, confirming that VL alleles may be protective. Following Bonferroni correction these associations were indicative of a trend, though not statistically significant.

### *TOMM40 ‘523’* alleles and progression to PDD in *APOE* ε3/ε3 carriers

Based upon PDD diagnosis, combined *‘523’* allele lengths were compared between PDD vs PD-ND groups. When gender was combined, a significant difference was observed between PDD and PD-ND (49.32 ± 14.42 and 51.33 ± 14.52, respectively; *p* = 0.036). When separated by sex, males with PDD and PD-ND exhibited no significant differences in combined ‘*523*’ allele length (49.28 ± 14.62 and 50.20 ± 14.95, respectively; *p* = 0.388). However, a significant difference in combined allele length was seen in females with PDD (49.42 ± 13.99) compared to females with PD-ND (53.25 ± 13.57; *p* = 0.043). While this association did not remain significant after Bonferroni correction (*p* = 0.172), it is suggestive of a protective effect of longer alleles.

Survival analysis using the Kaplan–Meier method, taking PDD diagnosis as the endpoints, showed no significant differences between S, L or VL carrier status when the sex of the PPMI cohort was combined (*p* = 0.396, *p* = 0.211 and *p* = 0.217, respectively). Cox proportional hazard regression models adjusting for sex also failed to show any significant associations (S allele, *p* = 0.485; L allele, *p* = 0.097; VL allele, *p* = .390). When considering males and females separately, and after excluding carriers of the L allele due to low numbers, no significant associations were observable between *‘523’* allele carrier status and progression to PDD in males (S allele, *p* = 0.587; VL allele, *p* = 0.720). Though no significance was seen in female carriage of the S allele (S allele, *p* = 0.618), a trend towards more rapid progression was observed in females lacking the VL allele (VL allele, *p* = 0.152).

## Discussion

Cognitive impairment and progression to PDD are significant determinants of morbidity and quality of life in PD. The genetic contribution to these non-motor aspects of PD is an area of great importance. Within studies examining cognitive decline in non-pathological ageing populations^19–22^ and in cohorts of individuals with AD^17,18^, the mitochondrial variant *TOMM40* ‘*523’* has been implicated with mixed results. By comparison, two studies have found no association between the *TOMM40* ‘*523’* and PD risk^32,33^, while a third group found that the L/VL ‘*523’* genotype was over-represented in Swedish PD patients when compared to controls^31^. However, research into whether *TOMM40 ‘523’* alleles play a role in modulating cognitive impairment and rate of decline within PD is profoundly scant, with only a single cross-sectional study of a mixed PD and DLB cohort reporting that *TOMM40 ‘523’* is not associated with risk of dementia^34^. To our knowledge, no previous studies have explored the relationship between *TOMM40 ‘523’* poly-T length variability and domain-specific cognitive decline or progression to dementia in PD, or differential effects of *TOMM40 ‘523’* alleles on cognition in males and females. As such, this is the first study to assess this relationship in a large, well-documented PD cohort undergoing serial cognitive assessments over a 5-year period. Our findings indicate that the *TOMM40 ‘523’* polymorphism does play a part in modulating cognitive decline, and suggest that alleles of *‘523’* have differential sex effects, with shorter alleles being associated with greater global cognitive decline in both males and females, whereas longer alleles have both global and domain-specific protective effects in females with PD.

In past literature, case–control analysis has found that while *TOMM40 ‘523’* does not modulate or influence the risk of developing PD, it may affect the age at which symptoms develop^32,33^. The findings of the present study provide evidence that polymorphism in *TOMM40 ‘523’* may also have significant effects on cognitive decline and progression to PDD, independent of the influence of *APOE* ε4. While our findings demonstrate an effect of *‘523’* on cognitive measures over time, significant differences were seen in the trajectory of cognitive decline and progression to PDD in females. Specifically, survival analysis showed a trend towards an association of S allele carriage with faster progression to PDD, whereas carriage of VL alleles was significantly protective in terms of progression to PDD in females, but not males. Furthermore, combined *‘523’* allele length was significantly shorter in females with PDD compared to those without dementia (PD-ND group).

In view of the linkage disequilibrium which exists between *TOMM40* and *APOE*, in order to accurately assess the independent contribution of *‘523’* allelic variation, *APOE* genotypes must be taken into account^16^. Thus, further analysis examined the sub-cohort of carriers of *APOE* ε3/ε3, which is the most common genotype and accounted for ~60% of the present PD cohort. The aforementioned associations in the whole cohort were reflected in the assessment-specific analysis of the sub-cohort of *APOE* ε3/ε3 carriers, when adjusting for covariates. For instance, S allele carriage was associated with greater decline in global cognitive ability (MoCA), as well as language fluency (measured by SFCOM assessment) and processing speed and attention (SDMT); while possession of VL alleles was protective of processing speed and attention. However, it is likely that these findings were also being driven by gender, as a number of significant associations between the *‘523’* variant and cognitive decline in females were not apparent in males. In particular, within the female *APOE* ε3/ε3 group, *‘523’* S allele carriage was associated with a significantly worse overall MoCA score, whereas VL alleles were significantly protective in terms of overall cognition (MoCA score), as well as visuospatial function, language fluency, and superior executive function and working memory. Moreover, in the *APOE* ε3/ε3 sub-cohort, no *‘523’* allele stratification was observable in males with PD, whereas VL alleles appeared to be associated with slower progression to PDD (though not statistically significant) in females. It is possible that with a larger sample size of *APOE* ε3/ε3 genotype carriers, this allele and sex association may be confirmed. Taken together, these findings suggest that increasing *TOMM40 ‘523’* allele length has a protective effect in terms of cognitive decline in people with PD, and this effect is more noticeable in females.

Interestingly, a shorter *TOMM40* allele length appeared to be a risk factor for deterioration in cognitive performance over time in this PPMI cohort. Such findings appear to be reflective of those found in a smaller, cross-sectional study by Lindqvist and colleagues^34^, though this group reported that both S and VL alleles exhibited a trend towards being associated with dementia in a mixed PDD and DLB cohort. Overall, such variable associations between *TOMM40 ‘523’* and aspects of cognition must be interpreted with caution. Furthermore, it is important to consider underlying differences between the two cohorts that might explain variation in findings, such as the age at disease onset, as a number of studies in the literature have suggested that an older age of onset is more likely to be associated with cognitive impairment^41–46^. Although information on age of onset was not provided in the Lindqvist paper^34^, their patient cohort appears to have been older than the PPMI cohort, which was comprised of recently diagnosed PwP with a mean age of onset of 59.9 years. The strengths of the present study are that it examined this relationship longitudinally in a large, homogeneous PD cohort, while considering the effect of the *APOE* ε4 carrier status, as well as the known variability of cognitive trajectory between males and females^40^. As such, the findings here support the notion that the *TOMM40 ‘523’* poly-T repeat variants do play a role in modulating cognitive decline in PwP, independently of the effects of *APOE* ε4.

As indicated by genetic studies, the role of mitochondrial dysfunction in PD aetiology and pathogenesis is increasingly well-recognised. A recent study consistently observed that TOMM40 protein deficits in brain samples from PwP correlated with a higher number of mitochondrial DNA deletions, and with enhanced oxidative stress, reduced ATP production and abnormal complex I protein concentrations^25^. Furthermore, it has been shown that TOMM40-facilitated importation of α-synuclein into mitochondria inhibits mitochondrial complex I^26^, a common source of mitochondrial dysfunction in PD. In addition to this, *TOMM40* is closely associated with the functioning of two familial PD genes, *Parkin* and *PINK1*, where TOMM40 is required by the PINK1 protein for mitochondrial localisation and Parkin recruitment in mitophagy^47^. Clearly, this variant may play a pathophysiological role in the disease risk and progression of PD. This, when combined with findings from studies that implicate the *TOMM40* variant in AD, cognitive decline in the healthy ageing and in DLB, is why such a variant is proposed as a region of interest in the context of PD-related cognitive ability.

Certain limitations of the present study should be noted. Firstly, as the PPMI cohort comprised only recently diagnosed PwP (with a mean disease duration of ~7 years and a mean age at onset of ~60 years), the present findings need to be confirmed in broader, more representative community-based PD cohorts. Moreover, as the cohort we examined was purely Caucasian, further studies are required to determine whether the reported allelic associations are also present in other racial and ethnic groups. In addition, given it has been suggested that using WGS for genotyping *TOMM40* ‘523’ may be less accurate than PCR-based techniques for longer alleles^48^, further studies should compare allelic associations using the two methods. Though the study was based on the use of well-recognised and validated cognitive instruments, future studies may benefit from utilising more comprehensive cognitive assessment protocols or modern imaging technology. Finally, despite being a comprehensive cohort, analyses of the L allele presented with power issues and, in some analyses, had to be excluded. Thus, future analyses using even larger comprehensive cohorts are warranted.

Although the role of the *TOMM40 ‘523’* poly-T repeat has been well-explored in AD and healthy age-related cognitive decline, there is a paucity of studies investigating whether it has a role in cognitive decline in PD. The findings of the present longitudinal study in a large PD cohort from the PPMI provide the first evidence that the poly-T repeat length is a significant determinant of cognitive decline, independent of *APOE4*, and that the effects are sex-dependent. Thus, while short *TOMM40 ‘523’* alleles were associated with more severe cognitive decline in both sexes, in females, longer alleles were associated with protective effects on global cognition, as well as particular cognitive sub-domains, including attention and processing speed, language fluency, working memory. and executive and visuospatial functions, and appeared to delay progression to PDD. Our findings highlight the importance of the *TOMM40 ‘523’* polymorphism in cognitive decline in PD and suggest that further studies in other PD populations would be worthwhile. When combined with sex and *APOE* genotype, *TOMM40 ‘523’* may be a viable prognostic marker, and may assist in understanding the pathology underlying cognitive decline in PD.

## Methods

### Participant recruitment and clinical evaluation of PD

The study considered 423 de novo PwP from the PPMI study cohort, out of which successful *‘523’* genotyping was carried out in 368 individuals (see section “*TOMM40 ‘523’* alleles and cognitive performance in *APOE* ε3/ε3 carriers”). Following this, as *‘523’* allele frequencies have been shown to be ethnic-specific^49^ all individuals categorised as Black, Asian, Hispanic/Latino or other were excluded to ensure a more homogenous Caucasian cohort. Data were collected longitudinally from January 2011, with six 12 monthly time points being considered (baseline, 1 year, 2 years, 3 years, 4 years and 5 years). All data pertaining to the PPMI cohort were obtained from the PPMI database (available at http://www.ppmi-info.org/data) on 1st November 2020. For up-to-date information on the study, visit http://www.ppmi-info.org. Inclusion criteria and more specific details of the PPMI study can be found in a previously published communication^50^.

Detailed demographic information was collected from all participants, including age at symptom onset, age at current assessment, disease duration and level of educational attainment. Anti-Parkinsonian medication dosage was obtained from the PPMI database as a total levodopa equivalent daily dose. All subjects were comprehensively evaluated, with motor symptom severity being assessed using the Movement Disorder Society-Unified Parkinson’s Disease Rating Scale) Part III, and disease severity on the Hoehn and Yahr scale^51^. The research involving human data was assessed and approved by The University of Western Australia Human Research Ethics Committee (approval number: RA/4/20/4293 and RA/4/20/4470). Written informed consent was obtained from all participants, visit http://www.ppmi-info.org for more information.

### Cognitive testing

Clinical and cognitive information utilised was also obtained from the PPMI database (available at http://www.ppmi-info.org/data). The neuropsychological assessments performed on PD subjects have been described previously^52^. Briefly, the PPMI utilised (i) the MoCA as a measure of global cognitive function (assessing working memory, visuospatial abilities, attention, language, orientation and executive function; score range: 0–30 points); (ii) the HVLT which measures episodic verbal memory (whereby 12 words are learned in triplicates and then a delayed recall is assessed); (iii) the BJLO, which is a measure of visuospatial function (whereby five practice items assessing spatial perception and orientation are followed by a testing period); (iv) the SFCOM to assess verbal fluency (multiple verbal fluency tests, being vegetable, fruit and animal fluency, are combined to assess verbal and lexical fluency); (v) the LNS test for executive function and working memory (in this test verbal working memory is tested by repeating a string of random letters and numbers to the assessor in ascending and alphabetical order); and (vi) the SDMT to assess processing speed and attention (this test uses geometric figures and numbers in a matching task)^53,54^. More detailed information regarding the cognitive assessments can be obtained at https://www.ppmi-info.org/study-design/research-documents-and-sops/. Published norms were applied for each of the various assessments^55–59^.

Finally, the diagnosis of PDD was made by the referring PPMI investigators, based on criteria developed by the MDS;^1,60,61^ and including information obtained from patients and family members, and cognitive impairment as defined by impaired performance (>1.5 SD below the mean) in at least two domains of one or more cognitive tests, together with evidence of functional impairment in everyday life as a result of cognitive dysfunction.

### *TOMM40* rs10524523 and *APOE* ε genotyping

Whole-genome sequencing data for the cohort of 423 cases were obtained from the PPMI database (available at http://www.ppmi-info.org/data), in order to genotype the rs10524523 (*‘523’*) risk allele in the PPMI cohort. As previously described^33^, binary alignment map files were aligned to the human reference genome GRCh38 using the Burrows-Wheeler transform alignment algorithm, and were analysed using the Integrative Genomics Viewer^62^, in order to calculate the length of the poly-T repeat, as previously demonstrated^48^. Repeat lengths were called by four separate investigators, and were checked for reproducibility. In samples where inter-rater differences existed, a fifth independent experienced investigator was conferred with, and when differences could not be reconciled that case was excluded. As a result, an unequivocal *‘523’* genotype was successfully determined in 368 of the 423 participants. Following this, individual poly-Ts were grouped as follows using the convention established by Roses et al.^18^: short (S, ≤19 Ts), long (L, 20–29 Ts) and very long (VL, ≥30 Ts). In addition, allele lengths in each individual were combined to calculate a total allele length. The *APOE* ε genotype was obtained from the PPMI database (available at www.ppmi-info.org/data), which was determined by the PPMI using TaqMan genotyping assays (Applied Biosystems Assay-On-Demand part numbers C_3084793_20 and C_904973_10)^63^, and involved the 7900HT Sequence Detection System (Applied Biosystems). Allelic and genotypic distributions of *TOMM40 ‘523’* and *APOE* variants in the cohort of 368 PwP are presented in Supplementary Table 1, and frequencies of *TOMM40* alleles relative to *APOE* allele carriage can be found online, as has been previously reported^33^. Each distribution was compared to distributions of European cohorts on the Webstr database, as previously reported^33^. Levene’s Test of Equality of Variances returned no significant differences, and observable similarities were discernible in the *‘523’* allele distribution of cohorts. Thus, the current cohort were considered reflective of a population of European descent and within Hardy-Weinberg equilibrium.

### Statistical methods

The PPMI cohort was analysed using IBM-SPSS software (version 26, IBM Corporation). A significant nominal *p* value < 0.05 was employed for all statistical tests. Variables were described using mean and standard deviation (SD), or frequency and percent (%), as appropriate. Normality was assessed using the Shapiro-Wilk test, with subsequent clinical characteristics analysed using independent samples *T* test, Mann–Whitney *U*, Kruskal-Wallis or Chi-square, as appropriate.

Within longitudinal studies, trends of mean clinical assessments and patient clinical characteristics were assessed over time using GLMMs. Unadjusted GLMMs were used as univariate models to assess if clinical characteristics were significantly associated with cognitive examination scores over time. Variables identified as being statistically significant in univariate models, as well as *APOE* ε4 status, were considered covariates and were included in multivariable adjusted GLMMs. Variables included in multivariable models as covariates included years between assessments, age at assessment, age at disease onset, disease duration, gender, years of education and *APOE* ε4 status (0, 1 or 2 copies). For determining the presence of multicollinearity, the variance inflation factor (VIF) was calculated for all independent variables. For all reported models, VIF values were <2. Adjusted GLMMs were constructed to assess the impact of *‘523’* genotype on cognition over time, independently of other covariates. As multiple comparisons were made in this exploratory study, we report both unadjusted *p* values, covariate-adjusted *p* values and covariate adjusted with Bonferroni corrected *p* values for each analysis. Bonferroni corrected *p* values were calculated by multiplying the *p* value to reflect the level of multiple hypotheses tested in this manuscript, being four levels. Furthermore, Akaike information criterion was used to compare model fit, where all multivariable models had lower values than unadjusted models, indicating better model fit. Residual plots were examined for all models and no violations were noted.

To evaluate the association between *TOMM40 ‘523’* genotype and progression to PDD, participants were stratified into PDD (dementia) or PD-ND (no dementia) groups, and mean combined *‘523’* allele lengths were compared between these groups and survival curves were estimated by the Kaplan–Meier method. To compare the survival curves, the log-rank test was applied, placing weight on longer survival periods^64,65^. In addition, all distributions of ages at PDD onset were compared via Cox proportional hazard regression models, while adjusting for *APOE* ε4 status. All aforementioned statistical analyses were also carried out when separating the cohort by sex, which is previously reported to be a significant factor in cognitive impairment within PD^40^ and in the sub-group of *APOE* ε3/ε3 carriers^16^.

## Supplementary information

Supplementary Information

## Data Availability

The full PPMI dataset, used in the preparation of this article, were obtained from the PPMI database (www.ppmi-info.org/data) and can be obtained following a short application process. For up-to-date information on the study, visit www.ppmi-info.org. Furthermore, any data pertaining to this article and not published within this article may be requested through collaboration.

## Code availability

No computer coding were used in the completion of the current manuscript.
