# Supplementary material for: TOMM40‘523’ poly-T repeat length is a determinant of longitudinal cognitive decline in Parkinson’s disease
Source: NPJ Parkinsons Dis. 2021 Jul 7;7:56. doi: 10.1038/s41531-021-00200-y (PMC8263775; doi:10.1038/s41531-021-00200-y)
Supplement: Supplementary file 1 — Supplementary Information [file 41531_2021_200_MOESM1_ESM.pdf]

SUPPLEMENTARY INFORMATION

Supplementary Table 1. Frequencies of *TOMM40 '523'* and *APOE* ε genotype and allele groupings (n=368).

	**Genotype/Allele**	**Frequency** n (%)
***TOMM40* '523' Genotype**	*S/S*	74 (20.1)
	S/L	40 (10.9)
	S/VL	145 (39.4)
	L/L	3 (0.8)
	L/VL	33 (9.0)
	VL/VL	73 (19.8)
***TOMM40* '523' Allele**	*S*	333 (45.2)
	L	79 (10.7)
	VL	324 (44.0)
***APOE* ε Genotype**	*ε2/ε2*	1 (0.3)
	ε2/ε3	56 (15.2)
	ε2/ε4	6 (1.6)
	ε3/ε3	205 (55.7)
	ε3/ε4	90 (24.5)
	ε4/ε4	10 (2.7)
***APOE* ε Allele**	*ε2*	64 (8.7)
	ε3	556 (75.5)
	ε4	116 (15.8)

Data presented as n (%). S, short; L, long; VL, very long; ε2, Apolipoprotein epsilon 2; ε3, Apolipoprotein epsilon 3; ε4, Apolipoprotein epsilon 4.

Supplementary Table 2. Longitudinal clinical characteristics of the PPMI cohort (n=368).

Clinical characteristics		Mean (SD) or n (%) Baseline (n=368)	Year 1 (n=342)	Year 2 (n=326)	Year 3 (n=320)	Year 4 (n=304)	Year 5 (n=276)
Age at assessment (yrs)		61.79 (9.56)					
Age at symptom onset (yrs)		59.85 (9.73)	59.76 (9.77)	59.69 (9.77)	59.65 (9.76)	59.33 (9.83)	58.99 (9.78)
Disease duration (yrs)		6.76 (6.65)					
Sex	Male	241 (65.5)	224 (65.5)	213 (65.3)	209 (65.3)	200 (65.8)	184 (66.7)
	Female	127 (34.5)	118 (34.5)	113 (34.7)	111 (34.7)	104 (34.2)	92 (33.3)
LEDD (mg/day)		-	303.97 (234.83)	399.76 (322.05)	497.16 (451.22)	572.48 (442.99)	708.28 (763.66)
H&Y		1.57 (0.50)	1.74 (0.52)	1.77 (0.54)	1.84 (0.54)	1.91 (0.62)	1.95 (0.55)
MDS-UPDRS III		20.92 (8.94)	23.03 (10.87)	22.73 (11.21)	23.94 (12.27)	24.21 (13.15)	24.53 (13.50)
Years of education (yrs)		15.55 (2.94)	15.48 (2.84)	15.61 (2.81)	15.65 (2.85)	15.66 (2.81)	15.67 (2.87)
MoCA	Total	27.22 (2.24)	26.53 (2.73)	26.49 (3.01)	26.52 (2.98)	26.58 (3.49)	26.75 (3.45)
HVLT	Total	24.46 (4.94)	23.97 (5.37)	23.95 (5.47)	24.83 (6.16)	24.04 (5.83)	24.67 (6.27)
BJLO		12.85 (2.07)	12.49 (2.28)	12.91 (2.11)	12.66 (2.19)	12.99 (2.00)	12.50 (2.21)
SFCOM		48.87 (11.76)	49.12 (11.53)	49.15 (13.21)	48.57 (11.98)	48.21 (12.53)	48.77 (13.09)
LNS		10.71 (2.62)	10.46 (2.68)	10.42 (2.79)	10.36 (2.98)	10.28 (3.20)	10.17 (2.94)
SDMT		41.08 (9.53)	40.89 (9.86)	40.22 (10.89)	40.28 (11.51)	39.33 (12.27)	40.03 (12.33)

LEDD, Levodopa Equivalent Daily Dose; H&Y, Hoehn & Yahr; MDS-UPDRS III, Movement Disorder Society-Unified Parkinson's Disease Rating Scale III; MoCa, Montreal Cognitive Assessment; HVLT, Hopkins Verbal Learning Test-Revised; BJLO, Benton Judgement of Line Orientation; SFCOM, Sematic Fluency test – COMbined; LNS, the Letter-Number Sequencing; SDMT, Symbol Digit Modalities Test.

Supplementary Table 3. Baseline clinical characteristics of the PPMI cohort (n=368).

Clinical characteristics		Mean (SD) or n (%) Combined (n=368)	Males (n=241)	Females (n=127)	Significance [a] (*p* value; Cohen's *d*)
Age at assessment (yrs)		61.79 (9.56)	62.26 (9.50)	60.91 (9.64)	*p*=.126; *d*=.14
Age at symptom onset (yrs)		59.85 (9.73)	60.44 (9.59)	58.73 (9.91)	*p*=.070; *d*=.18
Disease duration (yrs)		6.76 (6.65)	6.40 (5.99)	7.46 (7.72)	*p*=.422; *d*=.15
H&Y		1.57 (0.50)	1.57 (0.50)	1.57 (0.5)	*p*=.977; *d*=.00
MDS-UPDRS III		20.92 (8.94)	21.07 (9.05)	20.64 (8.75)	*p*=.757; *d*=.05
Years of education (yrs)		15.55 (2.94)	15.66 (2.90)	15.33 (3.01)	*p*=.255; *d*=.11
MoCA	Total	27.22 (2.24)	26.98 (2.23)	27.68 (2.20)	***p*=.001**; *d*=.32
HVLT	Total	24.46 (4.94)	23.57 (4.81)	26.14 (4.76)	***p*<.001**; *d*=.54
BJLO		12.85 (2.07)	13.20 (1.93)	12.18 (2.18)	***p*<.001**.; *d*=.50
SFCOM		48.87 (11.76)	46.37 (11.05)	53.67 (11.61)	***p*<.001**; *d*=.64
LNS		10.71 (2.62)	10.63 (2.64)	10.86 (2.60)	*p*=.360; *d*=.09
SDMT		41.08 (9.53)	40.01 (9.41)	43.12 (9.47)	***p*=.009**; *d*=.33

[a]Independent Samples test, Mann–Whitney *U* test or Spearman's Chi Square test conducted for between group analyses, based on sex.

LEDD, Levodopa Equivalent Daily Dose; H&Y, Hoehn & Yahr; MDS-UPDRS III, Movement Disorder Society-Unified Parkinson's Disease Rating Scale III; MoCa, Montreal Cognitive Assessment; HVLT, Hopkins Verbal Learning Test-Revised; BJLO, Benton Judgement of Line Orientation; SFCOM, Sematic Fluency test – COMbined; LNS, the Letter-Number Sequencing; SDMT, Symbol Digit Modalities Test.

Supplementary Table 4. Individual generalized linear mixed models assessing longitudinal association between participant cognitive scores and clinical characteristics (n=368).

Outcome	Model	Unadjusted				
		**Intercept**	**β-CoE**	**SE**	**t value**	**p^b value**
MoCA	Years between follow-up	26.965	-0.111	0.039	-2.868	**.004**
MoCA	Age at assessment	33.172	-0.101	0.006	-15.951	**<.001**
MoCA	Age at onset	32.765	-0.101	0.006	-15.954	**<.001**
MoCA	Disease duration	26.917	-0.018	0.009	-1.969	**.049**
MoCA	Male gender*	27.413	-1.003	0.136	-7.401	**<.001**
MoCA	LEDD	26.714	0.000	0.000	-1.903	.057
MoCA	MDS-UPDRS III	28.237	-0.064	0.006	-11.094	**<.001**
MoCA	Years of education	25.720	0.067	0.023	2.921	**.004**
HVLT	Years between follow-up	24.208	0.040	0.075	0.527	.598
HVLT	Age at assessment	38.050	-0.216	0.012	-17.840	**<.001**
HVLT	Age at onset	37.707	-0.225	0.012	-18.768	**<.001**
HVLT	Disease duration	24.442	-0.017	0.018	-0.934	.350
HVLT	Male gender*	25.989	-2.582	0.261	-9.875	**<.001**
HVLT	LEDD	24.475	0.000	0.000	-0.694	.488
HVLT	MDS-UPDRS III	26.780	-0.107	0.011	-9.506	**<.001**
HVLT	Years of education	20.871	0.219	0.044	4.940	**<.001**
BJLO	Years between follow-up	12.778	-0.011	0.029	-0.372	.710
BJLO	Age at assessment	15.097	-0.037	0.005	-7.414	**<.001**
BJLO	Age at onset	14.943	-0.037	0.005	-7.452	**<.001**
BJLO	Disease duration	12.930	-0.019	0.007	-2.757	**.006**
BJLO	Male gender*	12.171	0.882	0.101	8.708	**<.001**
BJLO	LEDD	12.789	-0.000	0.000	-0.642	.521
BJLO	MDS-UPDRS III	13.396	-0.027	0.004	-6.202	**<.001**
BJLO	Years of education	10.774	0.127	0.017	7.477	**<.001**
SFCOM	Years between follow-up	49.047	-0.112	0.165	-0.676	.499
SFCOM	Age at assessment	73.124	-0.381	0.027	-13.955	**<.001**
SFCOM	Age at onset	72.353	-0.395	0.027	-14.568	**<.001**
SFCOM	Disease duration	49.112	-0.035	0.040	-0.866	.387
SFCOM	Male gender*	53.973	-7.878	0.561	-14.049	**<.001**
SFCOM	LEDD	49.014	-0.001	0.001	-1.254	.210
SFCOM	MDS-UPDRS III	53.439	-0.198	0.024	-8.119	**<.001**
SFCOM	Years of education	39.075	0.623	0.097	6.417	**<.001**
LNS	Years between follow-up	10.641	-0.097	0.038	-2.547	**.011**
LNS	Age at assessment	17.638	-0.113	0.006	-18.460	**<.001**
LNS	Age at onset	17.178	-0.113	0.006	-18.541	**<.001**
LNS	Disease duration	10.394	0.004	0.009	0.470	.638
LNS	Male gender*	10.786	-0.539	0.136	-3.966	**<.001**
LNS	LEDD	10.307	0.000	0.000	0.012	.990
LNS	MDS-UPDRS III	11.539	-0.047	0.006	-8.167	**<.001**
LNS	Years of education	8.973	0.094	0.023	4.141	**<.001**
SDMT	Years between follow-up	41.052	-0.297	0.147	-2.017	**.044**
SDMT	Age at assessment	72.301	-0.501	0.023	-22.130	**<.001**
SDMT	Age at onset	69.761	-0.492	0.023	-21.711	**<.001**
SDMT	Disease duration	41.645	-0.135	0.035	-3.819	**<.001**
SDMT	Male gender*	43.077	-3.990	0.512	-7.792	<**.001**
SDMT	LEDD	40.471	-0.001	0.001	-1.210	.227
SDMT	MDS-UPDRS III	46.829	-0.273	0.022	-12.666	**<.001**
SDMT	Years of education	33.814	0.426	0.086	4.963	**<.001**

^{b}*p* value taken from GLMM without adjustment for covariates.

*Comparison category set to zero.

MoCA, Montreal Cognitive Assessment; HVLT, Hopkins Verbal Learning Test-Revised; BJLO, Benton Judgement of Line Orientation; SFCOM, Sematic Fluency test – COMbined; LNS, the Letter-Number Sequencing; SDMT, Symbol Digit Modalities Test; LEDD, Levodopa Equivalent Daily Dose; MDS-UPDRS III, Movement Disorder Society-Unified Parkinson's Disease Rating Scale III; β-CoE, beta Coefficient; SE, standard error; *p*, statistical significance (*p* value).

Supplementary Table 5. Capacity of *TOMM40* allele in predicting cognitive performance over time in males with PD, using unadjusted and adjusted generalized linear mixed models, in a subsection of *APOE* ε3/ε3 carriers within PPMI cohort (n=133).

**Model**	**Outcome**	**Unadjusted**					**Adjusted**				
		**Intercept**	**β-CoE**	**SE**	**t value**	***p*[b] value**	**Intercept**	**β-CoE**	**SE**	**t value**	***p*[c] value**
S Present *	MoCA	26.368	-0.738	0.240	3.075	**.002**	30.272	-0.602	0.235	-2.566	**.011**
	HVLT	23.498	-0.839	0.506	1.657	.098	28.837	-0.743	0.487	-1.523	.128
	BJLO	13.178	0.039	0.175	-0.224	.823	12.274	0.100	0.179	0.560	.576
	SFCOM	46.872	-2.000	1.016	1.968	**.049**	50.904	-2.023	1.033	-1.959	.051
	LNS	10.273	-0.153	0.247	0.619	.536	13.655	-0.141	0.242	-0.582	.561
	SDMT	38.667	-2.440	0.910	2.680	**.008**	55.368	-1.409	0.854	-1.650	.099
L Present *	MoCA	24.609	-1.911	1.633	1.170	.242	30.001	-0.453	1.558	-0.291	.772
	HVLT	20.584	-3.094	3.520	0.879	.380	28.466	1.448	3.271	0.443	.658
	BJLO	12.992	-0.179	1.380	0.130	.897	12.302	0.862	1.330	0.648	.517
	SFCOM	34.575	-12.739	7.302	1.745	.081	50.190	-6.477	7.156	-0.905	.366
	LNS	7.313	-3.000	1.766	1.699	.090	13.611	-1.245	1.645	-0.757	.449
	SDMT	23.519	-15.700	6.241	2.516	**.012**	54.957	-9.203	5.586	-1.648	.100
VL Present *	MoCA	26.525	0.033	0.207	-0.158	.875	30.356	-0.307	0.200	-1.537	.125
	HVLT	24.045	1.192	0.434	-2.745	**.006**	28.060	0.363	0.416	0.874	.383
	BJLO	13.158	-0.037	0.150	0.248	.804	12.518	-0.168	0.152	-1.107	.269
	SFCOM	47.011	-0.828	0.873	0.949	.343	52.127	-1.697	0.877	-1.935	.053
	LNS	10.414	0.344	0.212	-1.621	.106	13.550	0.035	0.206	0.168	.867
	SDMT	39.993	2.614	0.781	-3.346	**.001**	53.150	1.304	0.727	1.793	.073

[b]*p* value taken from GLMM without adjusting for covariates.

[c]*p* value taken from GLMM adjusting for covariates identified in Supplementary Table 4, including years between assessments, age at assessment, age at disease onset, disease duration, and years of education.

*Comparison category set to zero.

S, short; L, long; VL, very long; MoCa, Montreal Cognitive Assessment; HVLT, Hopkins Verbal Learning Test-Revised; BJLO, Benton Judgement of Line Orientation; SFCOM, Sematic Fluency test – COMbined; LNS, the Letter-Number Sequencing; SDMT, Symbol Digit Modalities Test; β-CoE, beta Coefficient; SE, standard error; *p*, statistical significance (*p* value).

Supplementary Table 6. Capacity of *TOMM40* allele in predicting cognitive performance over time in females with PD, using unadjusted and adjusted generalized linear mixed models, in a subsection of *APOE* ε3/ε3 carriers within PPMI cohort (n=72).

**Model**	**Outcome**	**Unadjusted**					**Adjusted**				
		**Intercept**	**β-CoE**	**SE**	**t value**	**p^b value**	**Intercept**	**β-CoE**	**SE**	**t value**	**p^c value**
S Present *	MoCA	27.782	-0.568	0.337	-1.683	.093	33.039	-0.905	0.327	-2.766	**.006**
	HVLT	26.338	-0.550	0.634	-0.868	.386	37.595	-0.917	0.584	-1.571	.117
	BJLO	12.165	-0.042	0.287	-0.148	.883	11.888	0.195	0.273	0.714	.476
	SFCOM	55.027	-1.843	1.320	-1.397	.163	66.591	-1.958	1.266	-1.546	.123
	LNS	10.462	0.379	0.311	1.221	.223	14.954	0.306	0.304	1.007	.315
	SDMT	46.229	-1.995	1.177	-1.695	.091	67.442	-1.815	1.081	-1.680	.094
L Present *	MoCA	27.353	-0.369	1.178	-0.313	.754	32.304	0.278	1.105	0.252	.802
	HVLT	25.990	-4.670	2.180	-2.142	**.033**	36.974	-3.733	1.945	-1.919	.056
	BJLO	12.109	1.412	0.982	1.438	.151	11.946	1.963	0.906	2.167	**.031**
	SFCOM	53.504	6.865	4.543	1.511	.132	64.582	9.266	4.185	2.214	**.027**
	LNS	10.737	1.069	1.072	0.997	.319	15.131	1.684	1.013	1.662	.097
	SDMT	44.724	-1.348	4.087	-0.330	.742	65.886	0.094	3.644	0.026	.979
VL Present *	MoCA	26.728	0.790	0.349	2.262	**.024**	31.452	1.024	0.329	3.110	**.002**
	HVLT	25.350	0.721	0.651	1.108	.269	36.079	0.912	0.585	1.560	.120
	BJLO	11.653	0.613	0.295	2.076	**.039**	11.598	0.542	0.275	1.969	**.050**
	SFCOM	50.582	3.849	1.341	2.870	**.004**	61.675	4.235	1.248	3.395	**.001**
	LNS	10.254	0.637	0.317	2.007	**.045**	14.633	0.687	0.302	2.273	**.024**
	SDMT	42.890	2.296	1.210	1.897	.059	63.924	2.543	1.085	2.345	**.020**

[b]*p* value taken from GLMM without adjusting for covariates.

[c]*p* value taken from GLMM adjusting for covariates identified in Supplementary Table 4, including years between assessments, age at assessment, age at disease onset, disease duration, and years of education.

*Comparison category set to zero.

S, short; L, long; VL, very long; MoCa, Montreal Cognitive Assessment; HVLT, Hopkins Verbal Learning Test-Revised; BJLO, Benton Judgement of Line Orientation; SFCOM, Sematic Fluency test – COMbined; LNS, the Letter-Number Sequencing; SDMT, Symbol Digit Modalities Test; β-CoE, beta Coefficient; SE, standard error; *p*, statistical significance (*p* value).

Supplementary Table 7. Longitudinal characteristics of the PDD and PD-ND groups.

Variable	Baseline		Year 1		Year 2		Year 3		Year 4		Year 5	
	PDD (n=7)	PD-ND (n=361)	PDD (n=33)	PD-ND (n=309)	PDD (n=49)	PD-ND (n=277)	PDD (n=66)	PD-ND (n=254)	PDD (n=58)	PD-ND (n=246)	PDD (n=43)	PD-ND (n=233)
Age	67.37 (7.60)	61.68 (9.57)	67.66 (8.00)	61.07 (9.59)	65.79 (8.07)	60.90 (9.75)	66.92 (8.21)	60.25 (9.53)	65.01 (8.39)	60.41 (9.84)	65.04 (8.08)	60.24 (9.72)
Male	7	234	22	202	38	175	52	157	47	153	35	149
Female	0	127	11	107	11	102	14	97	11	93	8	84
Disease duration	8.71 (6.70)	6.72 (6.65)	7.33 (5.71)	7.93 (6.88)	9.56 (7.07)	8.82 (6.80)	11.24 (7.48)	9.54 (6.63)	10.38 (5.60)	10.91 (7.07)	11.60 (6.43)	11.91 (6.94)
Years Education	16.57 (3.20)	15.53 (2.94)	15.15 (3.68)	15.51 (2.74)	14.92 (3.22)	15.73 (2.72)	15.76 (2.95)	15.62 (2.83)	15.38 (2.77)	15.72 (2.82)	15.49 (2.62)	15.71 (2.92)

PDD, Parkinson’s disease dementia; PD-ND, Parkinson’s disease no dementia
